# Statistical Approach to Incorporating Experimental Variability into a Mathematical Model of the Voltage-Gated Na^+^ Channel and Human Atrial Action Potential

**DOI:** 10.3390/cells10061516

**Published:** 2021-06-16

**Authors:** Daniel Gratz, Alexander J Winkle, Seth H Weinberg, Thomas J Hund

**Affiliations:** 1 Department of Biomedical Engineering, College of Engineering, The Ohio State University, Columbus, OH 43210, USA; gratz.24@osu.edu (D.G.); winkle.36@osu.edu (A.J.W.); weinberg.147@osu.edu (S.H.W.); 2 The Frick Center for Heart Failure and Arrhythmia, Dorothy M. Davis Heart and Lung Research Institute, The Ohio State University Wexner Medical Center, Columbus, OH 43210, USA; 3 Department of Internal Medicine, College of Medicine, The Ohio State University Wexner Medical Center, Columbus, OH 43210, USA

**Keywords:** computational model, electrophysiology, atrial fibrillation, arrhythmia, Bayesian statistics, voltage-gated sodium channel

## Abstract

The voltage-gated Na^+^ channel Na_v_1.5 is critical for normal cardiac myocyte excitability. Mathematical models have been widely used to study Na_v_1.5 function and link to a range of cardiac arrhythmias. There is growing appreciation for the importance of incorporating physiological heterogeneity observed even in a healthy population into mathematical models of the cardiac action potential. Here, we apply methods from Bayesian statistics to capture the variability in experimental measurements on human atrial Na_v_1.5 across experimental protocols and labs. This variability was used to define a physiological distribution for model parameters in a novel model formulation of Na_v_1.5, which was then incorporated into an existing human atrial action potential model. Model validation was performed by comparing the simulated distribution of action potential upstroke velocity measurements to experimental measurements from several different sources. Going forward, we hope to apply this approach to other major atrial ion channels to create a comprehensive model of the human atrial AP. We anticipate that such a model will be useful for understanding excitability at the population level, including variable drug response and penetrance of variants linked to inherited cardiac arrhythmia syndromes.

## 1. Introduction

Atrial fibrillation (AF) is the most common sustained arrhythmia in the U.S., and is associated with a range of comorbidities, including increased risk for heart failure and ischemic stroke. Unfortunately, the prevalence of AF is only increasing, while therapy has not advanced at the same pace, highlighted by the fact, that over the past 30 years, mortality rates for AF patients have actually increased [1]. Current therapies for AF face important limitations, including risk for procedural complications, efficacy, or adverse effects (e.g., ventricular arrhythmia for some pharmacological agents).

Voltage-gated Na^+^ channels are required for normal atrial excitability and defects in the function of the primary cardiac Na^+^ channel Na_v_1.5 have been linked to increased risk for AF. Furthermore, despite the risk for ventricular pro-arrhythmia, drugs that block Na_v_1.5 are commonly used in AF patients without structural heart disease [2,3]. Mathematical modeling has proven valuable in understanding the role of Na_v_1.5 in regulating cardiac excitability in normal and diseased states, including in the discovery and testing of novel drugs and therapies (e.g., CiPA project) [4,5,6,7].

In the context of AF, numerous atrial models have been developed with the purpose of further understanding the ionic basis of the disease and/or regional differences in excitability [8,9,10]. While these models have advanced our understanding of atrial cells both in health and disease, they have focused specifically on modeling an average or “typical” cell. Here we seek to propose a method for reparametrizing ion channels to better incorporate the natural variability between cells. Due to its central role in cardiac impulse generation and strong link to disease, we focus here on Na_v_1.5.

There has been a growing appreciation in the field for the fact that considerable heterogeneity exists in electrophysiological properties of cardiac myocytes even from the same heart/region, motivating a shift away from a modeling approach that focuses on recapitulating a single idealized myocyte towards an attempt to capture inherent variability in a representative population of cells. For the most part, populations of model cells have been generated by randomly perturbing the idealized parameters about the mean [11,12]. While this approach has been applied successfully to better understand drug effects and other population-level phenomena, it relies on several a priori assumptions about the important parameters and their variability in the population.

Here we apply a statistical approach to define a model of the voltage-gated Na^+^ channel that reflects heterogeneity in a population rather than a single idealized set of parameters. Specifically, we used a Markov chain Monte Carlo (MCMC) method from Bayesian statistics to find maximum a posteriori estimates for each biophysical parameter based on the available experimental data. This technique allowed us to fit statistical parameters involving the complex biophysical model, which would be intractable using more traditional approaches. By formulating the problem using statistical models, we could define a reasonable population of model fits in an empirical and rigorous manner.

Bayesian models have been used successfully to develop a wide range of biophysical models including action potentials, ion channels, calmodulin, and IP_3_ receptor [13,14,15,16,17]. Notably, Lei et al. used a MCMC approach to fit a model of hERG (*I*_Kr_) to hundreds of patch-clamp recordings simultaneously, which was then applied to comparing different models of temperature dependence of rate constants [18,19]. To our knowledge, our study represents the first effort to apply a similar approach to the voltage-gated Na^+^ channel Na_v_1.5. Beyond previous efforts [18,19], we also correct for inter-paper differences caused by temperature effects, as well as effects specific to labs and protocols. We apply this approach to a novel Na^+^ channel model, which is then incorporated into an established model of the human atrial action potential to compare emergent behavior in the context of the intact atrial myocyte. The resulting model was able to accurately reproduce variability in single cells as well as in fibers of cells. We find that there is substantial variability arising from the inter-paper differences, as well as intra-paper variability. We anticipate that the resulting model will be useful for studying population level responses to anti-arrhythmic drugs as well as understanding complex phenomena like variable penetrance of inherited ion channel defects.

## 2. Materials and Methods

### 2.1. Statistical Approach to Parameter Fitting for a Biophysical Model of the Voltage-Gated Na^+^ Channel Using Multiple Datasets

**Figure 1:**
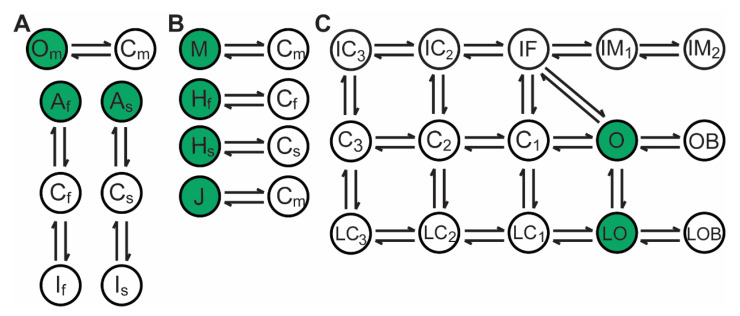
Examples of three different biophysical models of the volgate-gated Na^+^ (Na_v_) channel. Open/Active states are indicated in green, while closed/inactive states are white. (**A**) Our novel hybrid Na_v_ model with 3 independent components, an activation component (subscript *m*), and two inactivation components for fast and slow inactivation (subscripts *f* and *s*, respectively). The activation component consists of an open (*O*_m_) and closed (*C*_m_) state, while the inactivation components each consist of an active state (*A*), a closed state (*C*) and an inactivated state (*I*). (**B**) Example of a Hodgkin-Huxley type model consisting of 4 independent gating variables: activation (*M*), fast inactivation (*H*_f_), slow inactivation (*H*_s_) and recovery (*J*) [20,24]. (**C**) A Markov model where channel gating is represented as transitions between coupled states, including inactivation (I*X*), closed (C*X*), and open states (O). Additionally, there are further states to allow for late current (L*X*), and for drug block (*X*B) [22].

The ultimate goal of our study was to define a robust and physiological model of the human atrial voltage-gated Na^+^ channel (Na_v_) for use in computer simulations of human atrial excitability. While a variety of Na_v_ models have been published for use in simulations of the cardiac action potential (Figure 1), we proposed a novel formulation (Figure 1A) that sought to balance simplicity of Hodgkin-Huxley type models with channel gating assumed to occur via independent processes (Figure 1B) [20] and Markov Chain models in which gating is represented as transitions between dependent states (Figure 1C) [21,22]. Our resulting hybrid Na_v_ model featured separate Markov Chain models for distinct activation and inactivation processes, but grouped recovery into inactivation (details on model are provided in next section).

**Figure 2:**
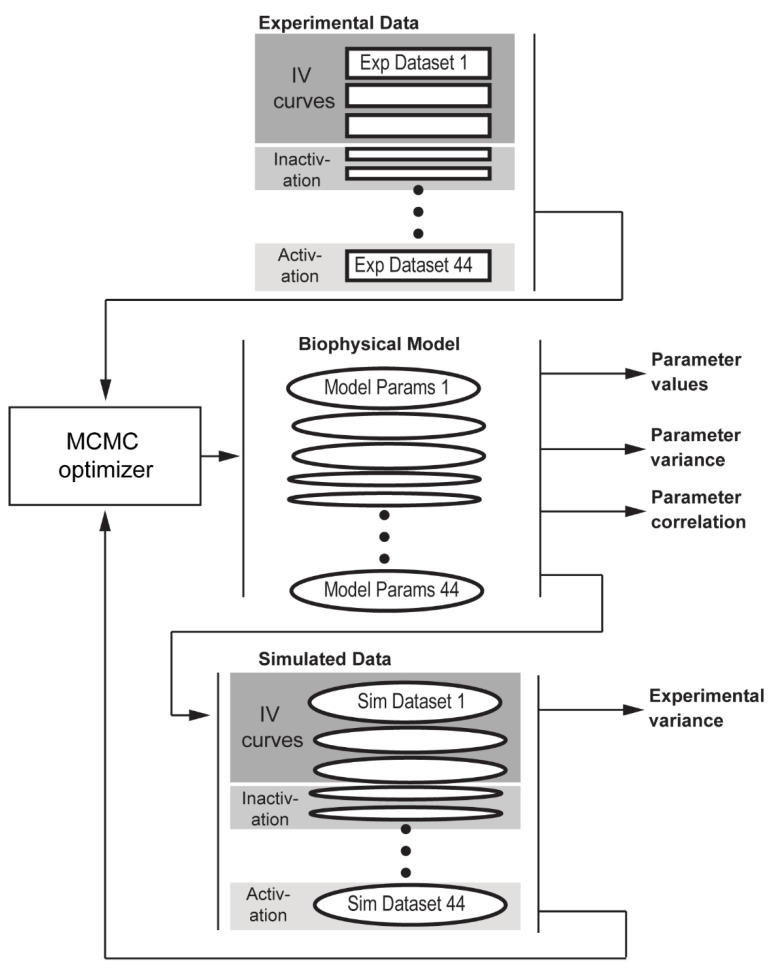
Statistical approach for fitting model parameters. Schematic illustrating the workflow used to fit biophysical model parameters using multiple sources of data. *Experimental Data* on voltage-gated Na^+^ channel measurements in human atrial cells are curated and provided as input to the statistical model fitting procedure [Monte Carlo Markov Chain (MCMC) optimizer)]. A subset of the data are held back for validation. The MCMC fitting procedure generates an initial guess for the set of parameters corresponding to each dataset, which are fed to the biophysical model to generate a set of *simulated results*. Independent normal variables with means of *simulated results* are compared to experimental data in the model to update the model fit while *experimental variances* encompass the variability between the predictions and experimental results and is used to track goodness of fit. Finally, the *Parameter Variance* and *Parameter Correlation* capture the variability between different experiments and the relationships between different parameters.

Having defined the structure of our model, the next step was to determine parameter values based on experimental recordings from human atrial myocytes published in the literature. Typically, a mathematical model of an ion channel is parameterized using data from a single source or average value, one parameter at a time. Although each experimental protocol aims to isolate a particular aspect of sodium channel behavior (e.g., steady-state inactivation or activation, peak conductance, kinetics), a single model parameter will likely influence results from several different protocols. Furthermore, this conventional approach is subject to bias by weighing results from one or few sources over others. To overcome these challenges, we parameterized our novel Na_v_ model using numerous sources with a range of experimental protocols and conditions to describe channel behavior. To facilitate simultaneous fitting of model parameters across multiple protocols and data sources, the Na_v_ model was embedded into a statistical model, which estimated the model error: the difference between the model’s predicted values for each experimental result (Figure 2). To account for different units corresponding to data collected using the different protocols, each result was allowed to have its own model error.

The statistical approach also allowed us to fit data from a number of different sources or publications. Naturally, different labs each with their own experimental conditions will measure different values, even when performing the same experiment (referred to here as the “undefined variability in experimental protocol effect” or UVEP effect). Accounting for this type of variability in a model is difficult to accomplish in either pre- or postprocessing steps. However, by embedding the Na_v_ model in a statistical model, variability due to the UVEP effect can be estimated during the model fit procedure. To accomplish this, we applied a concept from the ANOVA statistical test: the variable effect term, which provides an estimate for how different all the experiments in a given paper are from those in another paper. To ensure that the model was not simply fitting independent sets of biophysical model parameters to each paper, the following two constraints were added: (1) the effect term summed across all papers should approach 0; and (2) small UVEP effects are preferred over large UVEP effects. The first constraint ensures that there can be a meaningful set of parameter values that do not depend on the specific paper, while the second constraint prefers more parsimonious models. To impose the first constraint in a statistical model, we treated each UVEP effect as arising from a normal distribution with a mean of 0. The variance of this normal distribution was an additional parameter to be fit, and smaller variances were preferred, to apply the second constraint. Thus each UVEP effect is modeled as a random perturbation with a set variance. This approach is analogous to adding a variable effect term to an ANOVA model, for which a few samples are observed from a larger population.

A common experimental condition that varies between papers is the temperature of the bath used for current recordings. Although it would be possible to capture variability in this condition in the UVEP effect term, by treating it as an independent factor, we can use prior information from the literature to constrain dependence of model parameters on variable temperature [20,23].

### 2.2. Curation of Experimental Data from the Literature

A comprehensive literature review was performed to identify published studies including electrophysiological data from normal (non-diseased) human atrial myocytes. Data were converted to *json* format for storage and cross referenced in an excel spreadsheet with corresponding experimental conditions (Supplemental Table S1). Manual curation of the data was performed as quality control (i.e., physiological reversal potential for IV curve, activation/inactivation properties).

### 2.3. Mathematical Model of the Voltage-Gated Sodium Channel Na_v_1.5

Our novel hybrid Na_v_ model has 8 state variables that combines aspects of the first 2 approaches (Figure 1) with independent gating variables to represent activation and inactivation, similar to previous Hodgkin–Huxley models [20]. Consistent with previous models, the Activation module requires 2 state variables. Inactivation is separated into independent fast and slow components with each component represented by a 3-state Markov chain to allow for coupling of inactivation and recovery (not possible with pure Hodgkin-Huxley approach). For fast inactivation (and recovery) the Markov model consists of the *A*_f_ state for when the channel is Active; the *C*_f_ state for rapid Closing of the channel (comparable to the *h*_f_ gate in O’Hara–Rudy); and a further additional state for Inactivation of the channel *I*_f_ (analogous to the j gate in O’Hara–Rudy). Finally, as the inactivation module is split into fast and slow, there are 3 slow components (*A*_s_, *C*_s_, *I*_s_) complementary to the 3 fast components.

The model is parameterized by factors that are transformed into the model parameter from a factor value in -∞ to ∞, to a range which is valid for that specific model parameter. The first type of transformation is the exponential transformation for model parameters which can only take on values from 0 to ∞. An example of this is the conductance which has a base value of 1, $G_{Na}=1\cdot e^{G_{Na}Factor}$. Other model parameters that use the exponential transformation are the steepness parameters for rate constants or steady states and the maximum and minimum rates for a gate. The next kind of transformation is the logistic transform for model parameters that can only take on values between 0 and 1. There was only one parameter like this in the model, *A*_hf_, the proportion of fast vs. slow inactivation gates, $A_{hf}=\frac{1}{1+\;\exp\left(-A_{hf}Factor\right)}$. Finally, shifts to the locations of steady state curves or rate curves did not require any transformation as they could already take on any value from −∞ to ∞. For a full list of model parameters and their transformations see Table A1.

The *m* and *h* gates are parameterized separately. For steady state curves, logistic curves were used, while bi-exponential curves were used for rate constants (τ). For τ, a reparameterization was performed to reduce the dependence between its 4 parameters. Steady state and τ values were then transformed into Markov model rate constants, before they were used in the system of differential equations. For more details, see Appendix C.

The *m* gate had its own logistic steady state, *mss*, which was parameterized with a slope *mss_slope*, and the location of the midpoint *mss_shift*. *mss_slope* controls the steepness of the curve (0, ∞), with smaller values close to 0 being very gentle curves, while large values are very steep curves.

mss=11+exp(−(v+mssshift)mssslope )

The rate constant, τ, is the inverted rate from *O*_m_ to *C*_m_ (or the reverse direction) where large values indicate slow transitions and small values indicate fast transitions. τ was parameterized by the location of the maximum, the value at the maximum (τ_m_max_), the minimum asymptotic value (*baseline*) and the slopes of the exponentials to the right (τ_m_slope1_) and left (τ_m_slope2_) of the maximum.

τm=baseline15+τmmaxexp((v−τmshift)τmslope1)+exp(−(v−τmshift)τmslope2)

The *h* gate was split into a fast and slow component. The steady state curve was the same for both, but the τ‘s were different.

hss=11+exp((v−hssshift)hssslope)

The fast τ for *h* was parameterized as follows:

τhf=baseline10+τhfmax exp((v−τhfshift)τhfslope2)+exp(−(v−τhfshift)τhfslope1)

While the slow τ had a higher baseline, and different location, slope and maximum:

τhs=baseline+τhsmax exp((v−τhsshift)τhsslope2)+exp(−(v−τhsshift)τhsslope1)

Recovery was a part of the *h* gate, and so there is a recovery state in both the fast and slow components. However, the rates and steady states of recovery were the same for both components.

jss=11+exp((v−jssshift)jssslope)τj=baseline+τjmax exp((v−τjshift)τjslope2)+exp(−(v−τjshift)τjslope1)

The rate constants and steady states are then converted into Markov model transition rates, and the subsequent differential equations are solved. The solved gating variables are combined to find the Na_v_ open probability ($O_{m}^{3}\left[Ahf\cdot A_{f}+\left(1-Ahf\right)\cdot A_{s}\right])$. Where Ahf is the proportion of fast to slow inactivation. $O_{m}^{\;}$, $A_{f}$ and $A_{s}$ are the state variables from Figure 1A. The open probability is used in combination with the driving force and the conductance ($G_{Na}$) to compute the sodium current. For further details, see Appendix C.

### 2.4. Data Normalization

Before parameter fitting, experimental data were normalized depending on the experiment type. Briefly, Na^+^ current-voltage (IV) curves were split into two groups, those that had been normalized to cellular capacitance and those that had not. Data that were not normalized to capacitance were normalized to a minimum of −1 and then the signed square root ($sgn\left(x\right)\sqrt{\left|x\right|}$) was taken to reduce the influence of large values. Data that had been normalized to capacitance were not standardized to a specific minimum value and only the signed square root was taken. For all IV curves, the empirical reversal potential was compared to the expected reversal potential. If the difference was more than 10 mV, then the experiment was excluded. For smaller differences, the data was shifted to ensure consistency. Expected reversal potential was calculated using the Nernst equation which is provided in Appendix C. Steady state inactivation data were normalized to the value from the most negative voltage in the dataset, while activation data were normalized to range from 0 to 1. Time constants for both activation and inactivation were used together to produce simulated current traces that were fit, instead of fitting the time constants directly. Finally, recovery from inactivation data were normalized to the peak pre-pulse current, consistent with how these data are typically reported.

### 2.5. Bayesian Statistical Model Parameters

For estimation of the model parameters, as well as their variances, the Na_v_ model was embedded in a statistical model of the data (Figure 2). This enabled quantification of variability in and between datasets. Further, the statistical model can compensate for differences caused by temperature, or from different experimental procedures from different labs. A similar approach has been used previously to fit a potassium current from experimental recordings [19].

In our hierarchical model, the Results node compares model predictions with actual observed experimental values.

$$\begin{array}{c} X_{Results}=x_{biophysical}+\epsilon_{Experimental}\\ \epsilon_{Expiremental}\;\sim \;N\left(0,\;\sigma_{Experimental}^{2}\right) \end{array}$$

Results ($X_{Results}$) are the observed values (from published studies), which are modeled as the biophysical model’s prediction ($x_{biophysical}$) plus some experimental error/noise ($\epsilon_{Experimental}$). The experimental error was considered to be independent and distributed normally with mean 0, and a variance $\sigma_{Experimental}^{2}$. The variance depends on the specific experiment (e.g., measurement of IV curve, steady state inactivation, etc.), as different experiments have different magnitudes of error. For example, IV curves involve large values with a considerable amount of spread, whereas the steady state activation curve has much smaller values with less spread.

44 sets of parameters were used to define individual biophysical models for each experiment across all publications used for fitting (Figure 2). Each set of parameters contains 25 individual model parameters that are the specific parameters ($G_{Na}$, steady state parameters, rate parameters) that the biophysical model uses to predict outputs for experiment 1 through 44 ($x_{biophysical}$). Each set of parameters may be represented as $P=\left[\begin{array}{ccc} G_{Na}; & \ldots ; & \tau_{j\;tau1} \end{array}\right]$, and then combined into a matrix $PV_{i,j}=\left[\begin{array}{cc} P_{1} & \ldots \end{array}\right]$. Each row of the resulting matrix, i∈1…44, contains all the biophysical model parameters for an experiment, and each column, j∈1…25, represents the values for a specific biophysical model parameter (e.g., $G_{Na}$) over the range of experiments. Therefore, the value of each experiment P ($PV_{i}$ in the combined matrix) may be thought of as the following equation.

$$\begin{array}{c} P=\beta_{0}+\beta_{Temp}\cdot T+\sigma_{UVEP}^{2}+\varepsilon_{Parameter}\\ \varepsilon_{Parameter}\;\sim \;N_{25}\left(0,\;\Sigma_{Parameter}\right) \end{array}$$

where $\beta_{0}$ (intercept) is a 25-dimensional vector containing one value per parameter defining the mean value across all experiments; T is the difference in temperature from 17 °C; $\beta_{Temp}$ (temperature dependent effect) is a 25-dimensional vector defining the change in parameters with each additional 1 °C increase in temperature (T); $\sigma_{UVEP}^{2}$ (the UVEP effect) is a 25-dimensional vector defining the error caused by undefined variability in experimental procedures across labs; $\varepsilon_{Parameter}$ is the random variation between experiments and follows a multivariate normal distribution with mean 0 and covariance matrix $\Sigma_{Parameter}$, to allow for correlations between parameters. For the full details of the model, as well as the priors chosen, see Appendix A.

Taken as a whole, this statistical model produces a number of useful estimates, including: (1) the best biophysical parameters for a specific experiment, constrained by the group mean and variance; (2) the biophysical parameter mean while compensating for the temperature effect, and the influence of specific undocumented inter-study variability (UVEP effect); and (3) the variance of each parameter as well as its correlation with the other parameters. In a typical model fitting approach, the only estimate that would be created would be biophysical parameter mean without compensating for temperature dependence. These additional estimates allow for many further uses of the model. We will highlight two: (1) the temperature dependence allows for an improvement in estimating the mean; and (2) the parameter variance-covariance matrix can be used to generate a more realistic population of models.

### 2.6. Parameter Estimation

The estimates for each parameter from the Bayesian statistical model were computed jointly using MCMC to generate maximum a posteriori estimates, which maximize the likelihood of the model given the data. Initial values for the MCMC optimization procedure were assigned by the fitting library PyMC3 to be the mean of the prior distribution. Due to the heavy computational cost of repeatedly simulating all the experiments, only one chain could be run. For all parameters where a gradient could be computed, the NUTS (No-U-Turn Sampler) sampler was used as recommended by PyMC3 [25]. This encompassed all nodes in the model graph except $PV_{i,j}$, the model parameters for each experiment, as the gradient of the biophysical model is intractable. For $PV_{i,j}$ a Metropolis-Hastings (MH) step was used with a multivariate normal jump and the addition of a crossover step every 10 iterations.

The crossover step was performed independently for each set of parameters for an experiment. A subset of parameters was selected, independently with 10% probability. Then another experiment was selected at random from a predefined group, and the selected parameters in that experiment were used to replace the selected parameters in the current experiment. This step was then evaluated in the usual acceptance rejection fashion. The predefined groups which were allowed to exchange parameters with each other were either experiments from the same paper or of the same type.

To tune the MH step, an adaptive approach was taken, where the scaling of the jump size was updated to achieve the ideal rejection rate and the variance-covariance matrix of the jump distribution was updated using a moving average of the empirical variance-covariance of the chain [26]. Each experiment was tuned independently. The crossover step required no tuning.

### 2.7. Code

Model development and fitting were done in python version 3.7 using numpy 1.17 (1.18), pandas 0.25 (1.0), scipy 1.4, pymc3 version 3.8 (3.9) [27,28,29,30]. The code is available on github in hundlab/iNaCells2021Code, while the data is available in hundlab/iNaCells2021Data. Model testing and integration into the Grandi atrial myocyte was written in c++17, using the LongQt library 0.5. LongQt is available at hundlab.engineering.osu.edu/research/LongQt with model code available at hundlab/LongQt-model in branch atrial-model [31].

### 2.8. Simulations

Voltage clamp simulations were run according to the experimental procedures used to produce the data from the selected publications. The implicit backward-differentiation formula solver (BDF) from the scipy integrate library was used to solve the ordinary differential equations for the sodium channel model. The default values of relative and absolute tolerance were left at their defaults, 10^−3^ and 10^−6^, respectively.

For the single cell and 1D fiber simulations, a well-validated model of the human atrial action potential was used [8], with the original sodium current formulation replaced with our novel formulation. The simulation platform *LongQt* was used to run all cell and fiber simulations [31,32].

## 3. Results

### 3.1. Estimates of Na_v_ Model Parameters: Simultaneous Fitting of Values Corresponding to Individual Experiments and across the Population

An innovative aspect of our approach is that we are able to generate individual sets of parameters for specific experiments, while also fitting the overall parameter means and variances. In order to simulate each individual experiment from each paper, the protocol which was used in that experiment was replicated in the simulation and post-processed in a manner analagous to the actual experiment. For each individual protocol, a complete parameterization of the biophysical model was executed. From these individual fits, an overall average fit could then be compiled encompassing all experimental data. Further, the amount a parameter varies across individual fits was captured as an overall variance, as well as an overall correlation.

**Figure 3:**
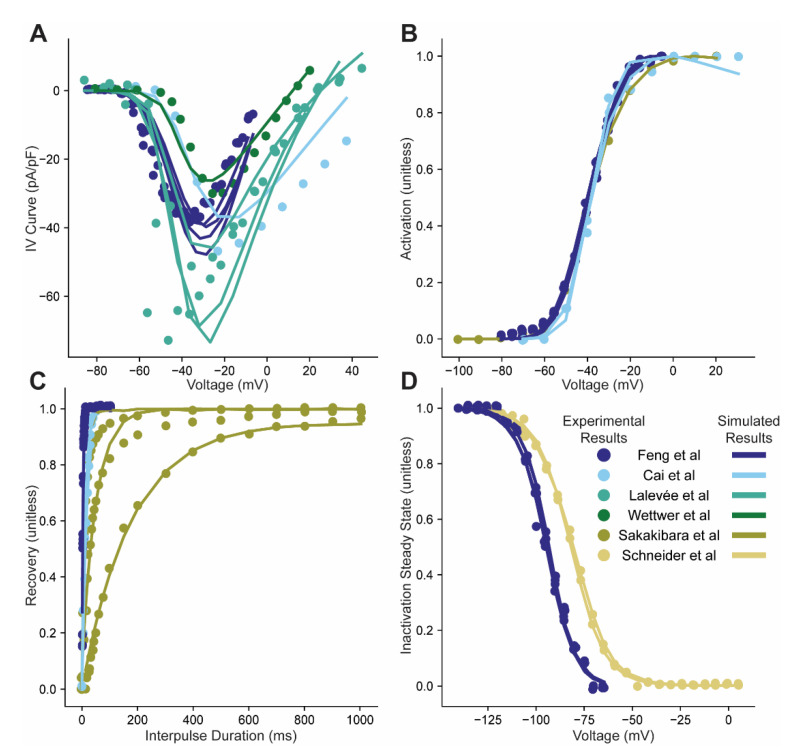
Simulated results of individual model fits to specific experiments. (**A**–**D**). Current Voltage relations, steady-state activation, recovery from inactivation, and steady-state inactivation curves using a different set of fit model parameters predicted by the statistical model (solid lines) for each experiment (circles). Data from Feng et al. had 4 experimental groups which were the lengths of time the cells were kept before an experiment was performed: 0, 1, 3, 5 days; Cai et al. had two experimental groups: cells taken from adult or pediatric patients; 3 control IV curve experiments were taken from Lalevée et al.; Wettwer et al. had a single IV curve control experiment; Sakakibara et al. had one control activation curve and three recovery curves with different inter-pulse holding potentials: −100, −120, −140 mV; and two inactivation curve experiments were taken from Schneider et al. with different durations for the inactivation potential: 512 and 256 ms [33,34,35,36,37,38]. Data from different experiments taken from the same paper are indicated with a single color indicated in the legend (same for corresponding simulation results).

The model was able to reproduce both the characteristic trends in the data as well as the individual differences between datasets (Figure 3). The model was able to reproduce different IV curves presented in the same paper, generated under similar or identical conditions, as well as IV curves reported in different papers (Figure 3A). Notably, location of the peak current for data from Lalevée et al. had a value between −40 and −50 mV, which our model predicts to be closer to −30 mV. This is not a case of the model being unable to fit the locations of these peak currents, instead there is a tradeoff between the best possible individual fit, and what is a reasonable fit given all the other observed data. In this case, this tradeoff pulls the estimates back towards the overall fit. There was also qualitative agreement between the simulated and experimentally measured steady-state activation curves, which, in general, showed less variability across papers (Figure 3B). There are a large range of time scales across publications for recovery curves, with Sakakibara et al. being much slower than Feng et al. or Cai et al. (Figure 3C) even after taking into account the different protocol’s holding potentials. Specifically, Sakakibara et al. used 3 different holding voltages from −100 mV which was the same as was used in Cai et al. to −140 which was the same as Feng et al. Nevertheless, the individual fits were capable of accurately reproducing these large differences between curves (taking into account differences in holding potentials). The inactivation steady state curves were quite similar from both datasets, and were all fit very precisely (Figure 3D).

**Figure 4:**
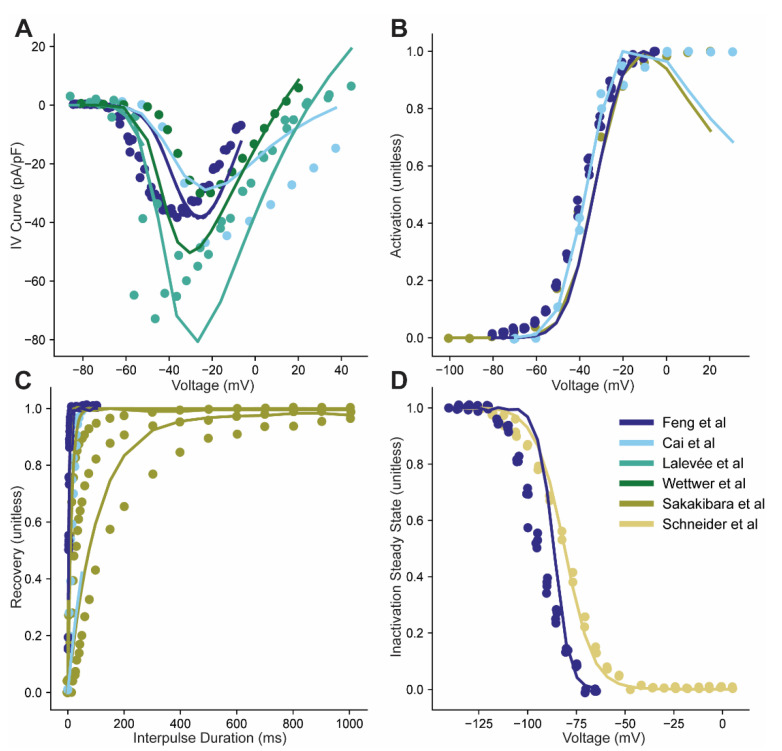
Simulated results of overall model fits to specific xperiments. (**A**–**D**). Current Voltage relations, steady-state activation, recovery from inactivation, and steady-state inactivation curves using a single set of fit model parameters with experiment-specific temperature and sodium concentrations reported for each study. Experimental data is the same as in Figure 3 from Feng et al., Cai et al., Lalevée et al., Wettwer et al., Sakakibara et al. and Schneider et al. [33,34,35,36,37,38]. Data from different experiments taken from the same paper are indicated with a single color indicated in the legend (same for corresponding simulation results). In the event that a different experimental protocol was used to measure the same property (e.g., recovery from inactivation) multiple simulation results are indicated with the same color corresponding to results using the same model parameters but different protocols.

Only the parameter fits to individual experiments are tested in the biophysical model during the fitting procedure. In contrast, the overall mean and variance of the model population is not directly tested in the biophysical model, but instead are extracted from the population of individual fits. A first test of the resulting fit from the statistical model is to determine how closely the overall fit extracted from the population of models fits the data. Therefore, we simulated the results of using the overall mean fit along with temperature effects to determine how well the mean generated by our population of individual models fit the mean predicted by the statistical model. Specifically, the UVEP effect was not used and will not be used going forward, so that the fits in Figure 4 and subsequent figures reflect only the overall fit. The UVEP effect could be reintroduced to simulate results based on data generated in an individual paper, however published differences in protocol or conditions (e.g., bath Na^+^ concentration) were implemented when available. Supporting success of the fitting procedure, the model using parameters from the overall fit, in general, produced simulated results consistent with the experimental data (Figure 4A–D). It is interesting to note that the greatest discrepancy between model and experiment could be observed for the location of the peak in the IV curve (Figure 4A).

**Figure 5:**
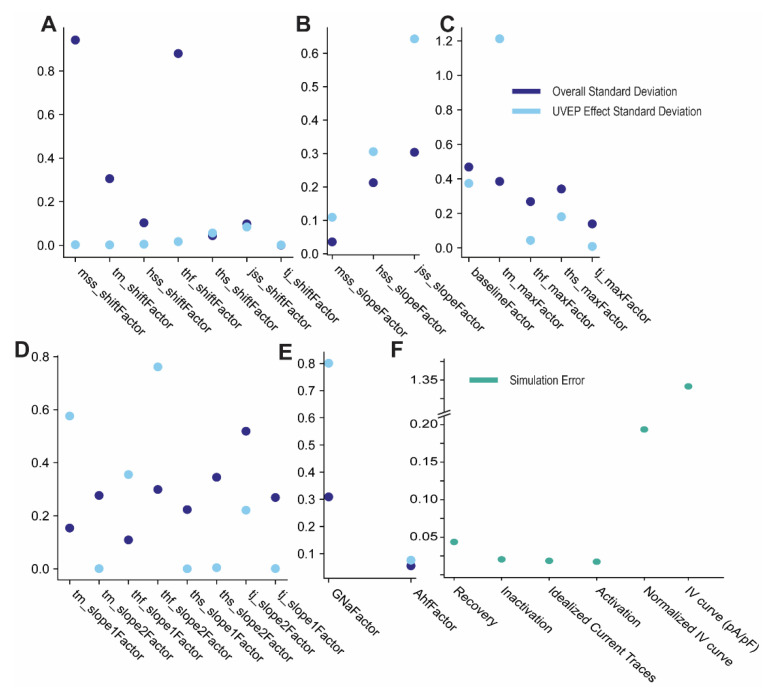
Fitting of model parameters using maximum a posteriori (MAP) estimates. (**A**–**E**) The overall standard deviations (dark blue) and the UVEP effect standard deviations (light blue) for each model parameter. Estimates for standard deviations were made using maximum a posteriori values that maximize the likelihood of the statistical model (Appendix A). Model parameter factors are grouped according to functionality. (**A**) Parameter factors responsible for locations of steady state and rate curves. (**B**) Parameter factors responsible for the steepness parameters of the steady state curves. (**C**) The minimum and maximum values of the rate constants. Baseline is scaled for each rate constant by constants ^1^/_15_, ^1^/_10_, 1, 1, for gates *m*, *h*_f_, *h*_s_, *j* respectively. (**D**) The steepness parameters for the rate constants. (**E**) The conductance, *G*_Na_, and the proportion of fast to slow inactivation. (**F**) The estimated standard deviations for the error components in the model. Each error term corresponds to a different type of experiment. IV curve simulation error has units pA/pF, while all other errors are for normalized measurements and are unitless.

Aside from the parameter mean values, the fitting procedure generates several different estimates for standard deviation, the overall standard deviation of the individual fits, the standard deviation of the UVEP effect, and the model error, which is the error the model makes in predicting the experimental results (Figure 5). The overall variability in the model parameters for shift of *m*_ss_ and *t*_h,f_ are by far the largest (Figure 5A); this is due specifically to the large range of shifts needed to fit the IV curves (Figure 3 and Figure 4). This is interesting, as it indicates that in order to shift the IV curve, it requires a shift in both parameters rather than just *m*_ss_ as might be expected. Further, it is actually the rate of inactivation that makes a difference, not the location of its steady state curve. While this might seem counterintuitive, this is because even though inactivation is much slower than activation, it is still fast enough to effect peak current. Thus, to shift the location of the IV curve both parameters must be changed, which also results in a positive correlation between the shifts of *m*_ss_ and *t*_h,f_ (Figure S2). As this variability in both *m*_ss_ and *t*_h,f_ was only present in the IV curve experiments, for future applications of the fitted model these variabilities were reduced to 0.4.

Our estimates of the variability in steepness of the steady state curves (Figure 5B), indicated that there is considerable variability both overall, and between papers. The variability between papers is consistently larger than the overall variability, which indicates that experimental conditions may play a large role in this variability. By contrast, the variability in the maximum time constants values (τ) (Figure 5C) is constantly higher for the overall standard deviation, with the exception of the *m* gate. This, along with large UVEP effects in the slopes of the τ (Figure 5D) suggests that the *m* gate may be strongly affected by variability in undocumented conditions across experiments/labs.

Upon estimating model errors (Figure 5F), we found that IV curves had substantially more variability than other experiments, even after they were normalized. The other experiment types had more similar degrees of simulation error. The range of error predicted across experiments supports the a priori decision to allow for separate error estimates.

The estimates of the correlations, showed moderate to weak associations between model parameters (Figure S2). This may be in part due to our reparameterization of the rate constants to reduce the dependencies between parameters, which is described in the Appendix B.

### 3.2. Generation of a Population of Na_v_ Models Based on Overall Parameter Fits

**Figure 6:**
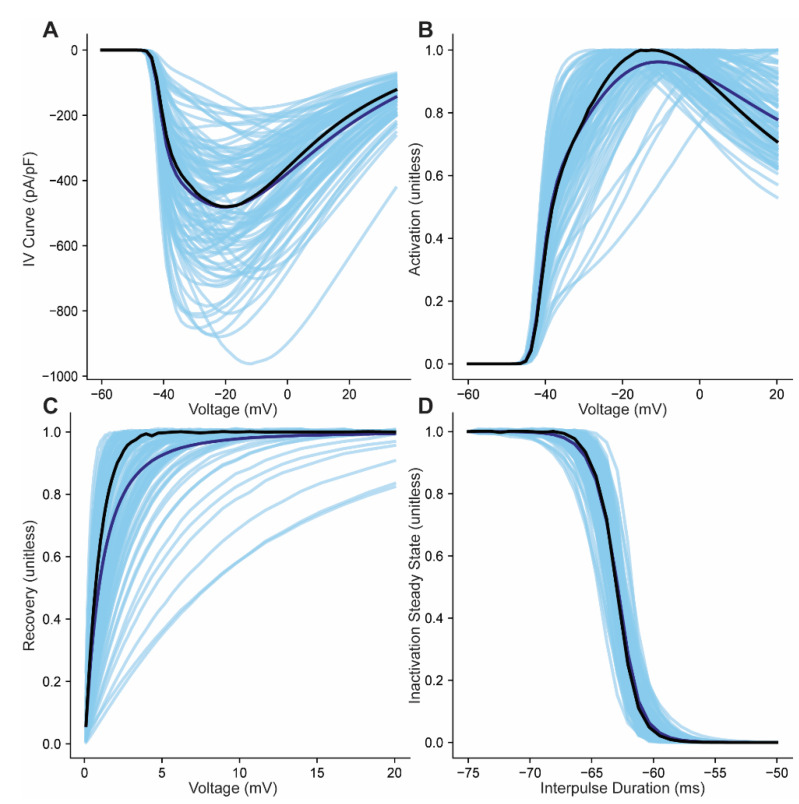
Simulation of model population at physiological temperature. (**A**–**D**) Current-voltage, steady-state activation, recovery from inactivation, and steady state inactivation curves from a population of models generated by randomly selecting 100 parameter sets from the fitted 25 dimensional multivariate normal distribution. The individual results from the 100 simulations (light blue lines) were used to generate a population mean (dark blue line) for each property and compared to the theoretical mean (black line) generated using the mean of the entire normal distribution. Simulations used the sodium concentrations and temperature used by the Grandi full sell AP model [9.1 and 140 mM, internal and external sodium, respectively, and temperature of 40 °C (unlike previous simulations (Figure 3 and Figure 4) that used room temperature to facilitate comparison to experiment (difficult to measure Na_v_1.5 at body temp due to its rapid kinetics and large amplitude)].

Our estimates of the variances and correlations are also highly important components of the model, as these define the physiological range of parameter values. To test if extracting new values (not used in the determining the overall fit) would result in physiological range of behavior, we drew 100 parameter combinations from the fitted 25-dimensional normal distribution, with the overall mean and temperature effect, and the overall variance covariance matrix. Next, we simulated the four different kinds of experiments shown previously with these randomly drawn values and examined the results. Unlike previous simulations (Figure 3 and Figure 4) that used room temperature to facilitate comparison to experiment (difficult to measure Na_v_1.5 at body temp due to its rapid kinetics and large amplitude), the temperature for these plots is set to body temperature. The resulting IV, activation, inactivation and recovery from inactivation curves show a distribution that is relatively tight (Figure 6A–D). The mean from this test population of 100 individual models compares well to the true mean of the distribution (curves are virtually superimposed in Figure 6), which validates that this population is similar to the data we used to fit the model during the fitting process.

### 3.3. Incorporation of Na_v_ Model into a Comprehensive Model of the Human Atrial Action Potential

**Figure 7:**
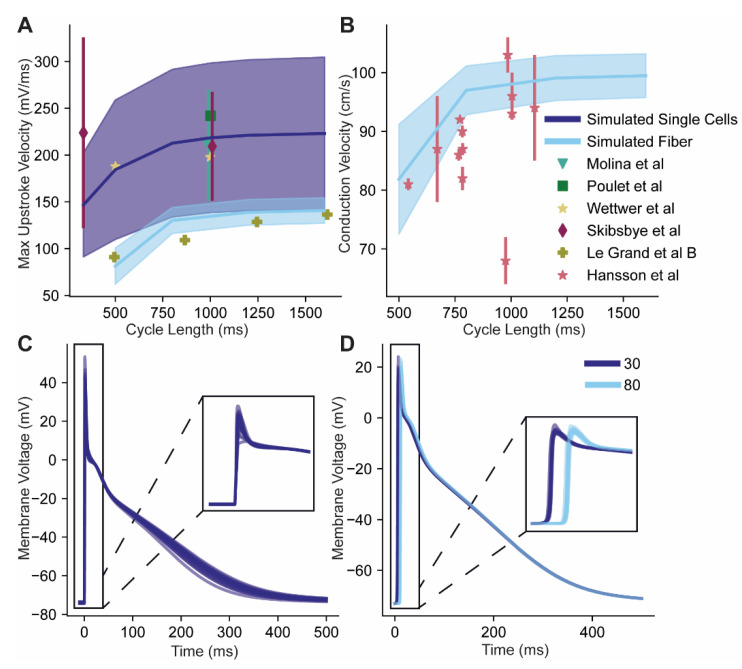
Model validation. The new voltage-gated Na^+^ channel model was incorporated into a detailed model of the human atrial action potential [8]. Validation for single cell dynamics was performed by comparing the maximum upstroke velocity and conduction velocity at different cycle lengths to published data, in single cells and in fiber. (**A**) Solid dark blue line is the mean, and the dark blue shaded region is the standard deviation for the maximum action potential upstroke velocity from the generated population of 100 cells. Similarly, the light blue line and shaded region are the mean and standard deviation, respectively, from a population of 40 fibers, each of which is 100 cells long. For the fiber, the upstroke velocity of the fiber is calculated as an average of cells, $X_{i}$, from the middle of the fiber: $X_{20},\;X_{30},\;\ldots ,\;X_{70},\;X_{80}$. Solid dots are values reported in literature and error bars are standard deviations. Model was tuned by hand to the mean of Molina et al. (**B**) Conduction velocity, light blue line and shaded region use the same fibers as in A. Conduction velocity was calculated using the 30th and the 80th cells. Data from Hansson et al. is on individuals, so each point represents one individual. (**C**,**D**) Sample traces from individual cells and from cells in the fiber, respectively. Data was taken from [36,40,41,42,43,44].

As a final validation step for our model and parameterization approach, we incorporated the fitted Na_v_ model into a whole cell human atrial myocyte model [8]. Importantly, the data used in validation were not used to fit the model, and only one dataset came from a paper that was also used in the model fitting process. Some adjustments needed to be made to fit the model in the whole cell: the location of the inactivation steady state curve had to be moved in the positive direction by 16 mV. This is likely a result of the steady state curve datasets being exceedingly negative, as has been commented on previously [39]. Next, the slopes of the activation and inactivation steady state curves needed to be increased by 1.6. Finally, the conductance was tuned so that the resulting mean was close to the mean value from Molina et al. (Figure 7). Notably, the variability seen in Molina et al. was not used in the tuning process.

Action potential upstroke velocity was measured in the model, both in simulated single cell pacing, as well as in a 100 cell fiber (Figure 7). For single cell simulations, all of the observed data falls comfortably within the 1 standard deviation band (Figure 7A). Further, the 1 standard deviation band in both Molina et al. and Skibsbye et al. is quite close to our simulated band, but is slightly more narrow. Resulting action potentials showed a physiological range of AP morphologies (Figure 7C). For the fiber of cells (to simulate, for example, a human atrial trabecula), there was only one paper with published data on upstroke velocity, with observations divided into two groups: one which was highly triangular AP labeled group A (not shown), and the other which had a plateau phase and was labeled group B. For our comparison, we choose to use group B as they had a typical shape of healthy APs, while highly triangular APs have commonly been associated with disease. There was one additional study that quantified conduction velocity in multiple individuals (Figure 7B). For these measurements on healthy cells, our fiber measurements align with the observed data, and show a similar response to changes in basic cycle length (Figure 7A,B, action potentials from individual cells in the fiber shown in Figure 7C).

## 4. Discussion

### 4.1. Bayesian Modeling Provides a Natural Way to Incorporate Different Data into One Model

When developing a new model it is advantageous to use as much data as possible to ensure that the model has the opportunity to learn how the system operates in a diverse set of conditions. This presents a distinctive challenge for conventional approaches as these larger datasets are often only obtainable by compiling data from different publications from different labs, with different protocols, etc. To compensate for all of these complicating factors thus necessitates more sophisticated models and fitting procedures. We propose that a natural path forward is to borrow concepts and models from statistical literature, namely Bayesian statistical literature, and embed the biophysical model of interest in a larger statistical model. A similar approach has been used before to measure cell-to-cell variability, and has shown this approach’s effectiveness for fitting the hERG channel in high-throughput systems [19]. Our work shows that statistical modeling can also be effective using data from traditional patch-clamp systems and the more complex Na_v_.

By embedding the biophysical model in a statistical model, we can combine the strengths of both approaches. The biophysical model is capable of capturing the complex time and voltage relationships seen in the Na^+^ current in myocytes in a concise and interpretable fashion, which is why they have been at the core of electrophysiological modeling for almost 70 years. They also have the ability to help compensate for some highly important factors such as changes in the voltage clamp protocol, or changes in sodium ion channel concentrations, which vary across experiments and labs. On the other hand, it is difficult to capture other changes in the environment in the biophysical model, such as the effect of temperature. This is often not an issue in simulating myocytes, as they also operate at a fixed temperature. However, for fitting Na_v_, this provides a particular challenge, as the experiments are typically done at room temperature, while the system of interest naturally operates at body temperature. To complicate the situation, ambient temperature can vary considerably across studies, so some of the available data may be taken at one temperature while the rest is at a different temperature. Incorporating this information into the fitting process is non-trivial; however, this is possible in the statistical model. Further, one uniquely Bayesian component is that some information from other publications can be incorporated directly into the model. In the case of temperature, we used the results of a different publication to characterize its effect, rather than trying to extract that from our primary dataset.

It is worth noting that we found numerous large UVEP effects, suggesting that there are important differences between papers, which are not explicitly defined in the experimental protocol or the model. These differences may be the result of preparation techniques and solution; artifacts in different patch clamp systems; heterogeneity in myocyte populations, or heterogeneity across individuals [45]. Further exploration of the roots of this variability could yield important insights into the many factors which influence myocytes and the sodium channel in particular.

Despite large UVEP effects and significant variability within papers, the average parameter fits (Figure 4) seem to be reasonable summaries of the individual fits (Figure 3). While the ability to average data together is often taken for granted in cardiac research, it is not guaranteed that an average will be representative of the individual points which were averaged together. This fact has been noted in neuronal tissue, where averaging sometimes fails, and is associated with the measured properties having complex associations with one another [46]. Our work suggests that in the context of Nav averaging is in fact a valid approach and should yield summaries which are in fact representative of the underlying data.

One of the key components of Bayesian statistical models is their ability to incorporate information known previously about the problem into the statistical model, through prior distributions. One example of this is how we used previous information on temperature effects to inform its effect in the model. More broadly, however, prior distributions provide a powerful tool to incorporate the results of previous fits into future work. For example, if more data was gathered on the sodium channel in human atrial myocytes, the results from this study could inform prior distributions for the model of the new data. The new model would then fit the new data, but be biased toward the results from this study; that is, it would find a compromise between the new data and the old data. This is powerful in the sense that the new fit is still informed by the old data, without needing to actively fit to all of the old datasets. This effect also scales such that as more new data is present, the old data becomes “washed out” and has a weaker and weaker effect on the model. Thus Bayesian modeling provides powerful tools and intuitive approaches to handling disparate data.

Another powerful feature of a Bayesian statistical model like the one we have described is that an individual experiment does not need to conclusively define all the parameters needed to specify the biophysical model. As long as some experiment does define that parameter, it will be defined in the overall summaries. For example, an IV curve is not significantly affected by the parameter values that control recovery from inactivation. Thus, in a traditional approach, it would be impossible to fit the model to individual experiments and instead only an overall fit across all the experiments would be obtainable. However, in our approach, the overall mean and variances are fit at the same time, and so the overall estimates can both infer parameter values from experiments where that parameter is well defined and impose realistic restrictions on the experiments where that parameter is poorly defined. Together, they allow us to estimate the collected set of experiments as a population, rather than as one overall mean.

### 4.2. Variability Is of the Next Frontier for Electrophysiological Modeling

Having a variety of data is even more crucial when incorporating variability, as this shifts the focus from finding one average fit of the data to finding a population of fits to the data. Modeling a population also requires a model that can describe that variability, which is by necessity a statistical model. Bayesian statistical models fit this requirement and have the additional desirable property that they can interface with complex non-statistical models, so long as the parameter space can be explored efficiently. This is in contrast to other common statistical models, which typically require all model components to be highly tractable.

We believe that modeling variability is an important next step in electrophysiological modeling. As models continue to be used for increasingly important tasks such as drug screening, it is crucial that we ensure the population of models we are using truly represent the underlying population of interest. Previous approaches have mostly introduced variability into a model by assuming a distribution with defined mean and standard deviation. In this conventional approach, parameters (usually various conductances) are assigned a sampling distribution and then independent and identical samples are draw for each parameter many times to form a population. While this is a reasonable first approximation and an efficient way to generate a population of models, there is no guarantee that the sampling distribution is physiologically meaningful. This may not be a significant issue, especially when the source of variability is confined for example to heterogeneity in ion channel conductances where high throughput sequencing can provide reasonable constraints. However, the challenge becomes greater as more sources of variability are considered (for example, differences in channel gating). Going forward, we expect that the many uses of models will require increasing accuracy and confidence in their prediction, which cannot be met without modeling of variability. Further we have shown that a statistical modeling framework can provide such estimates of variability from a complex dataset, and in one of the most complex ion channels in the heart. We expect that the techniques described here will play an invaluable roll in characterizing the variability in other ion channels and in a full myocyte model.

The techniques applied here may also be combined with more conventional approaches to find a balance between simplicity and accuracy. Depending on the subject of interest, some ion channels may not be as critical to producing cell models with realistic variability. In these cases, it would be effective to vary the less important ion channels using only the conductance with a simple distribution. Then by using this more in depth model for the more critical ion channels, the resulting population may be very good approximation of the true population without needing to fit every ion channel using our new approach.

### 4.3. Limitations

There are many sources of variability which can contribute to the overall and UVEP standard deviations and range from artifacts or errors in the patch clamp measurements to differences in experimental preparation to myocyte heterogeneity across tissue regions and human populations. We have divided this variability into the two groups (overall and UVEP) in order to better understand the impact of these sources at these two different levels in the data. However, these data are not fine-grained enough to facilitate a more detailed modeling of all of these potential sources, and so it is certainly possible that some of the variability seen in the overall standard deviation is not physiological in nature, but is instead the result of experimental artifacts [45].

We have shown that a statistical approach may be used to generate a physiological population of Na_v_ and action potential models using heterogeneous data from multiple (but limited) sources. The greatest challenges in this approach arise from the complexity of the Na_v_ model and the low information content of the individual experiments in the dataset. The Na_v_ channel is inherently complex [47], both in terms of the number of parameters needed to define the model as well as in finding numerical solutions to its differential equations. The large number of parameters leads to a huge search space that a fitting algorithm must traverse in order to find reasonable solutions, resulting in long fitting times. Additionally, computing the numerical solutions to each experiment is compounded by the number of simulations needed to reproduce the >500 points from 44 experiments. This connects to what we mean by the low information content of each experiment. In other words, to reproduce, for example, an IV curve with 20 data points, 20 separate simulations are needed, and only the peak current is used from each simulation. This means that the model must simulate a huge amount of data, most of which is thrown out, simply to represent a handful of points. Thus more than >500 simulations needed to be run at each step of the fitting procedure. While all simulations could be executed in < 30 seconds, the overall effect was that the total fitting time required was in excess to 200 computational hours utilizing a 20 core processor.

We expect that for more complex models, such as the L-type calcium channel, or very large Markov models, this approach would be difficult to implement effectively. Similarly, for whole cell models, the fitting times would be very large. However, these challenges could be partially or fully ameliorated by modeling more directly the experimental recordings, as they would provide a much richer dataset, and thus would require fewer simulations to be run at each step in the fitting process. Further, for simpler ion channels such as the potassium channels, the size and computational expense of the models are greatly reduced and would make excellent targets for this approach.

## Data Availability

Data sharing not applicable.

## Supplementary Materials

The following are available online at https://www.mdpi.com/article/10.3390/cells10061516/s1, Figure S1: Normalized IV Curves and Current Traces, Figure S2: Correlation Matrix, Table S1: Experimental Conditions.

## Appendix A. Full Statistical Model

The statistical model used by the fitting procedure for maximum a posteriori estimation.

$$\begin{array}{c} X_{Results,j}=x_{biophysical,j}+\epsilon_{Experimental,j,l,m}\\ \epsilon_{Expiremental,j,l,m}\;∼\;N\left(0,\;\sigma_{Experimental,l}^{2}\right)\\ \sigma_{Exerimental,l}∼InverseGamma\left(0.001,\;0.001\right)\\ x_{biophysical,j}=BiophysicalModel\left(PV_{j}\right)\\ PV_{i,j}={\left[\begin{array}{c} P_{i} \end{array}\right]}_{j}\\ P_{i}=\beta_{0,i}+\beta_{Temp,i}\cdot T_{k}+\mu_{UVEP,i,\;k}^{2}+\varepsilon_{Parameter,i,j}\\ \beta_{0,i}∼N\left(\mu_{\beta_{0\;},i},\;\sigma_{\beta_{0},i}^{2}\right)\\ \beta_{Temp,i}∼N\left(\mu_{\beta_{Temp},i},\;\sigma_{\beta_{Temp},i}^{2}\right)\\ \mu_{UVEP,i,\;k}∼N\left(0,\;\sigma_{UVEP,i}^{2}\right)\\ \varepsilon_{Parameter,j}\;\sim \;N_{25}\left(0,\;\Sigma_{Parameter}\right)\\ \sigma_{UVEP,i}^{\;}∼InverseGamma\left(\alpha_{prior,i},\;\beta_{prior,i}\right)\\ \Sigma_{Parameter}=diag\left(\sigma_{Parameter}\right)\;\rho_{Parameter}\;diag\left(\sigma_{Parameter}\right)\\ \sigma_{\mathrm{Parameter}}∼InverseGamma\left(\alpha_{prior},\;\beta_{prior}\right)\\ \rho_{Parameter}∼Lewandowski˗Kurowicka˗Joe\left(\eta =1\right) \end{array}$$

The detailed coefficients for the priors are provided below. Most priors were vague but proper, except for $\beta_{Temp}$ where temperatures effects were defined or for *Ahf*. Information on *Ahf* was given in two publications (Sakakibara et al. and Cai et al.) used for fitting, however incorporating this directly into the experimental data was not always possible and the small amount of data present was not sufficient to constrain *Ahf*, thus we choose to restrict the priors, setting *Ahf* to have a mean of 0.85 in accordance with the results from those publications [34,37].

μβ0,i={0: i∈0,…, 17, 19, …,241.7:i=18σβ0,i={100: i∈0,…, 17, 19, …,24110:i=18μβTemp,i={−7100: i∈1,4,10,14,21410:i∈6,9,11,15,20,22−410:i=30:otherwiseσβTemp,i={1100: i∈1,4,6,9,10,11,14,15,18,20,21,22100:otherwiseαprior, i={0.001: i∈0,…, 17, 19, …,2411:i=18βprior,i={0.001: i∈0,…, 17, 19, …,241:i=18

i∈0…24, The biophysical model parameter, names provided in Table A1 below.

j∈0…44, The experiment, see Supplementary Table S1 for individual experiments and their protocols pages: iv_curve and gating.

k∈0…5, The paper, fully determined by j, the experiment number.

l∈0…5, The experiment type, fully determined by j, the experiment number.

$m\in 0\ldots n_{j}$, The index of an individual data point; $n_{j}$, The number of data points in the *j*th experiment.

In addition to the priors a potential was also added to the model to reduce the impact of the idealized current traces, as they were artificial data, and thus provided more data points than the other kinds of experiments. The loglikelihood for each idealized current traces was divided by the square root of the number of artificial points in the current trace.

**Table A1:** The biophysical model parameter.

Index	Transformation	Model Parameter Factors
0	$G_{Na}=exp\left(G_{Na}Factor\right)$	$G_{Na}Factor$
1	baseline=2.038 exp(baselineFactor)	baselineFactor
2	$mss_{slope}=9.871\;exp\left(mss_{slope}Factor\right)$	$mss_{slope}Factor$
3	$mss_{shift}=51.57+mss_{shift}Factor$	$mss_{shift}Factor$
4	$\tau m_{max,inital}=0.474\;exp\left(tm_{max}Factor\right)$	$tm_{max}Factor$
5	$\tau m_{slope1}=34.77\;exp\left(\tau m_{slope1}Factor\right)$	$\tau m_{slope1}Factor$
6	$\tau m_{shift,inital}=-57.6+tm_{shift}Factor$	$\tau m_{shift}Factor$
7	$\tau m_{slope2}=5.955\;exp\left(tm_{slope2}Factor\right)$	$\tau m_{slope2}Factor$
8	$hss_{slope}=14.086\;exp\left(hss_{slope}Factor\right)$	$hss_{slope}Factor$
9	$hfss_{shift}=-76+hss_{shift}Factor$	$hss_{shift}Factor$
10	$\tau hf_{max,inital}=5exp\left(\tau hf_{max}Factor\right)$	$\tau hf_{max}Factor$
11	$\tau hf_{shift,inital}=-57.64+thf_{shift}Factor$	$\tau hf_{shift}Factor$
12	$\tau hf_{slope1}=6.285\;exp\left(\tau hf_{slope1}Factor\right)$	$\tau hf_{slope1}Factor$
13	$\tau hf_{slope2}=15\;exp\left(\tau hf_{slope2}Factor\right)$	$\tau hf_{slope2}Factor$
14	$\tau hs_{max,inital}=5.894\;exp\left(\tau hs_{max}Factor\right)$	$\tau hs_{max}Factor$
15	$\tau hs_{shift,inital}=-66.947+ths_{shift}Factor$	$\tau hs_{shift}Factor$
16	$\tau hs_{slope1}=28.05\;exp\left(\tau hs_{slope1}Factor\right)$	$\tau hs_{slope1}Factor$
17	$\tau hs_{slope2}=40\;exp\left(\tau hs_{slope2}Factor\right)$	$\tau hs_{slope2}Factor$
18	$Ahf=\frac{1}{1+exp\left(-AhfFactor\right)}$	AhfFactor
19	$jss_{slope}=20\;exp\left(jss_{tau}Factor\right)$	$jss_{slope}Factor$
20	$jss_{shift}=-110+jss_{shift}Factor$	$jss_{shift}Factor$
21	$\tau j_{max,inital}=100exp\left(\tau j_{max}Factor\right)$	$\tau j_{max}Factor$
22	$\tau j_{shift,inital}=-80+tj_{shift}Factor$	$\tau j_{shift}Factor$
23	$\tau j_{slope2}=10\;exp\left(\tau j_{slope2}Factor\right)$	$\;\tau j_{slope2}Factor$
24	$\tau j_{slope1}=20\;exp\left(\tau j_{slope1}Factor\right)$	$\tau j_{slope1}Factor$

## Appendix B. Equation Reparameterization

The inverted bi-exponential was reparameterized to allow control over the location and value of the peak. These transformations were performed before the biophysical equations in Methods, which parameterized the rate constants for gates *m*, *h*, and *j*, as in the following equation.

$$\tau x=\frac{\tau x_{max}}{\exp\left(\frac{v-\tau x_{shift}}{\tau x_{slope1}}\right)+\exp\left(\frac{-\left(v-\tau x_{shift}\right)}{\tau x_{slop2}}\right)}$$

However the $\tau x_{max}$ and $\tau x_{shift}$ were modified as below.

τxshift=shiftadjust−τxshift, initalτxmax=τxmax,adjust·τxmax,initalshiftadjust=log(τxslope1τxslope2)1τxslope1+1τxslope2τxmax,adjust=exp(shiftadjustτxslope1)+exp(−shiftadjustτxslope2)

In these equations, τx is the rate constant of a gate and v is the transmembrane voltage. The parameters which we adjust are: $\tau x_{max}$ the maximum value of the curve; $\tau x_{shift}$ the voltage at which the maximum ($\tau x_{max}\;$) occurs. Without these modifications, the location and value of the maximum are dependent on the values of $\tau x_{slop1}$ & $\tau x_{slop2}$, so it was necessary to first calculate the maximum height correction $\tau x_{max,adjust}$ and the shift correction $shift_{adjust}$, in order to find values of $\tau x_{max}$ and $\tau x_{shift}$, which would correspond to a maximum at $\tau x_{max,inital}$ with its location at $\tau x_{shift,\;inital}$. Before these adjustments the max and location would change with different slope parameters. The advantage of this different parameterization of the inverted bi-exponential is that max and its location follow an intuitive definition than the parameters in other parameterizations. Further, this allows for the maximum and its location to be independent of each other and the slopes. The slopes, however, cannot be made entirely independent from one another. Reducing the dependence of variables on each other improves the efficacy of the fitting process.

## Appendix C. Markov Model

The rate constants and steady states defined in Methods are converted into Markov model transition rates *a* and *b*.

$$\begin{array}{c} a=\left(\begin{array}{cccc} \frac{mss}{\tau m} & \frac{hss}{\tau h_{f}} & \frac{hss}{\tau h_{s}} & \frac{jss}{\tau j} \end{array}\right)\\ b=\left(\begin{array}{cccc} \frac{\left(1-mss\right)}{\tau m} & \frac{\left(1-hss\right)}{\tau h_{f}} & \frac{\left(1-hss\right)}{\tau h_{s}} & \frac{\left(1-jss\right)}{\tau j} \end{array}\right) \end{array}$$

These transition rates are then used to define the 3 independent sets of differential equations needed for the model. See Figure 1A for a state diagram of these equations.

$$\frac{d}{dt}\left[\begin{array}{c} O_{m}\\ C_{m} \end{array}\right]=\left[\begin{array}{cc} -b_{1} & a_{1}\\ b_{1} & -a_{1} \end{array}\right]\left[\begin{array}{c} O_{m}\\ C_{m} \end{array}\right]\;\frac{d}{dt}\left[\begin{array}{c} A_{f}\\ C_{f}\\ I_{f} \end{array}\right]=\left[\begin{array}{ccc} -b_{2} & a_{2} & 0\\ b_{2} & -\left(a_{2}+b_{4}\right) & a_{4}\\ 0 & b_{4} & -a_{4} \end{array}\right]\left[\begin{array}{c} A_{f}\\ C_{f}\\ I_{f} \end{array}\right]\;\frac{d}{dt}\left[\begin{array}{c} A_{s}\\ C_{s}\\ I_{s} \end{array}\right]=\left[\begin{array}{ccc} -b_{3} & a_{3} & 0\\ b_{3} & -\left(a_{3}+b_{4}\right) & a_{4}\\ 0 & b_{4} & -a_{4} \end{array}\right]\left[\begin{array}{c} A_{s}\\ C_{s}\\ I_{s} \end{array}\right]$$

Finally, after solving these systems of differential equations, the sodium current can be computed using the following equation.

$$\begin{array}{c} I_{Na}=G_{Na}O_{m}^{3}\left[Ahf\cdot A_{f}+\left(1-Ahf\right)\cdot A_{s}\right]\left[v-v_{rev}\right]\\ v_{rev}=\frac{R\cdot T}{F}\;\log\left(\frac{na_{o}^{+}}{na_{I}^{+}}\right) \end{array}$$

In this equation, *v* is the transmembrane voltage, *R* is the gas constant, *T* is the temperature in Kelvin, *F* is faraday’s constant, $na_{o}^{+}$ is the external sodium concentration, $na_{I}^{+}$ is the internal sodium concentration and $v_{rev}$ is the reversal potential for sodium. $G_{Na}$ and Ahf are the model parameters for conductance and proportion of fast to slow *h* gate, respectively.
