# Supplementary material for: Statistical Approach to Incorporating Experimental Variability into a Mathematical Model of the Voltage-Gated Na+ Channel and Human Atrial Action Potential
Source: Cells. 2021 Jun 16;10(6):1516. doi: 10.3390/cells10061516 (PMC8234464; doi:10.3390/cells10061516)
Supplement: Supplementary file 1 [file cells-10-01516-s001.zip › supplement final.pdf]

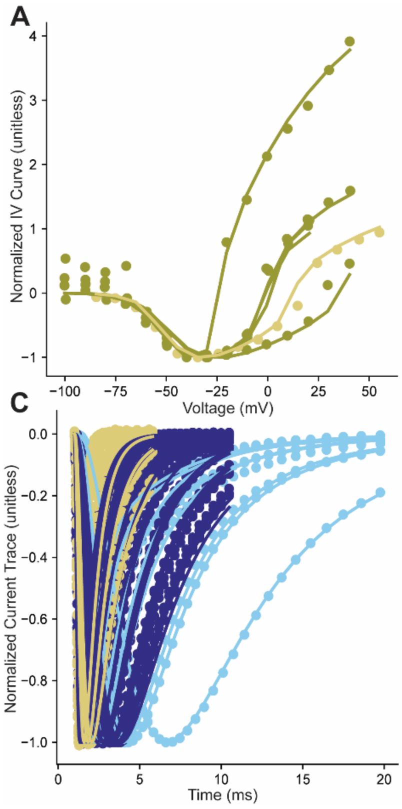

Supplementary Figure 1. Normalized IV Curves and Current Traces. Individual fits and overall fits for normalized IV curves and current traces. **A.** Individual fit for normalized IV curve. 5 experiments come from Sakakibara *et al.*, 2 of which are controls, and three of which are at varying external sodium concentrations: 2, 5, 20mM. 1 experiment comes from Schneider *et al.* which is a control. **B.** Overall fit for the same data. **C.** Individual fit for current traces. There are 7 experiments in this plot, 2 from *Cai et al.*, 4 from *Feng et al.*, and 1 from Schneider *et al.* Each experiment involved measuring activation and inactivation at multiple stimulation voltages, and so there are many current traces for each experiment. The 2 experiments from Cai *et al.* are from Adult and Pediatric cells; the 4 experiments from Feng *et al.* were kept for 0, 1, 3 and 5 days before experimentation; and the 1 experiment from Schneider *et al.* was a control. **D.** Overall fit for the same data. [1–4]

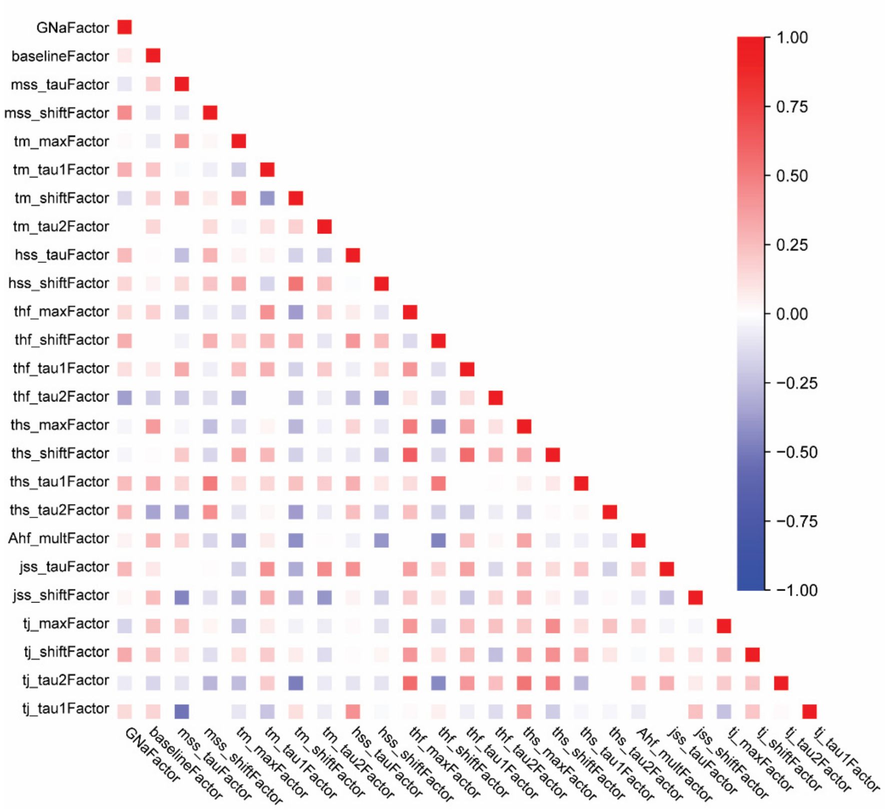

Supplementary Figure 2. Correlation Matrix. The correlations between the biophysical model parameters. Correlations were estimated based on the individual fits, in the same fashion as the overall variance, with one correlation between each pair of model parameters.

1. Sakakibara, Y.; Wasserstrom, J.A.; Furukawa, T.; Jia, H.; Arentzen, C.E.; Hartz, R.S.; Singer, D.H. Characterization of the sodium current in single human atrial myocytes. *Circ. Res.* **1992**, *71*, 535–546, doi:10.1161/01.RES.71.3.535.

2. Cai, B.; Mu, X.; Gong, D.; Jiang, S.; Li, J.; Meng, Q.; Bai, Y.; Liu, Y.; Wang, X.; Tan, X.; et al. Difference of Sodium Currents between Pediatric and Adult Human Atrial Myocytes: Evidence for Developmental Changes of Sodium Channels. *Int. J. Biol. Sci.* **2011**, *7*, 708–714, doi:10.7150/ijbs.7.708.

3. Schneider, M.; Proebstle, T.; Hombach, V.; Hannekum, A.; Rüdel, R. Characterization of the sodium currents in isolated human cardiocytes. *Pflügers Arch. Eur. J. Physiol.* **1994**, *428*, 84–90, doi:10.1007/BF00374755.

4. Feng, J.; Li, G.R.; Fermini, B.; Nattel, S. Properties of sodium and potassium currents of cultured adult human atrial myocytes. *Am. J. Physiol. Circ. Physiol.* **1996**, *270*, H1676–H1686, doi:10.1152/ajpheart.1996.270.5.H1676.
